# Use of plasma C-reactive protein, procalcitonin, neutrophils, macrophage migration inhibitory factor, soluble urokinase-type plasminogen activator receptor, and soluble triggering receptor expressed on myeloid cells-1 in combination to diagnose infections: a prospective study

**DOI:** 10.1186/cc5723

**Published:** 2007-03-16

**Authors:** Kristian Kofoed, Ove Andersen, Gitte Kronborg, Michael Tvede, Janne Petersen, Jesper Eugen-Olsen, Klaus Larsen

**Affiliations:** 1 Clinical Research Unit, Copenhagen University Hospital, Hvidovre, Kettegaard Allé 30, DK-2650 Hvidovre, Denmark; 2 Department of Infectious Diseases, Copenhagen University Hospital, Kettegaard Allé 30, Hvidovre, DK-2650 Hvidovre, Denmark; 3 Department of Clinical Microbiology, Copenhagen University Hospital, Blegdamsvej 9, Rigshospitalet, DK-2100 Copenhagen Ø, Denmark

## Abstract

**Introduction:**

Accurate and timely diagnosis of community-acquired bacterial infections in patients with systemic inflammation remains challenging both for clinician and laboratory. Combinations of markers, as opposed to single ones, may improve diagnosis and thereby survival. We therefore compared the diagnostic characteristics of novel and routinely used biomarkers of sepsis alone and in combination.

**Methods:**

This prospective cohort study included patients with systemic inflammatory response syndrome who were suspected of having community-acquired infections. It was conducted in a medical emergency department and department of infectious diseases at a university hospital. A multiplex immunoassay measuring soluble urokinase-type plasminogen activator (suPAR) and soluble triggering receptor expressed on myeloid cells (sTREM)-1 and macrophage migration inhibitory factor (MIF) was used in parallel with standard measurements of C-reactive protein (CRP), procalcitonin (PCT), and neutrophils. Two composite markers were constructed – one including a linear combination of the three best performing markers and another including all six – and the area under the receiver operating characteristic curve (AUC) was used to compare their performance and those of the individual markers.

**Results:**

A total of 151 patients were eligible for analysis. Of these, 96 had bacterial infections. The AUCs for detection of a bacterial cause of inflammation were 0.50 (95% confidence interval [CI] 0.40 to 0.60) for suPAR, 0.61 (95% CI 0.52 to 0.71) for sTREM-1, 0.63 (95% CI 0.53 to 0.72) for MIF, 0.72 (95% CI 0.63 to 0.79) for PCT, 0.74 (95% CI 0.66 to 0.81) for neutrophil count, 0.81 (95% CI 0.73 to 0.86) for CRP, 0.84 (95% CI 0.71 to 0.91) for the composite three-marker test, and 0.88 (95% CI 0.81 to 0.92) for the composite six-marker test. The AUC of the six-marker test was significantly greater than that of the single markers.

**Conclusion:**

Combining information from several markers improves diagnostic accuracy in detecting bacterial versus nonbacterial causes of inflammation. Measurements of suPAR, sTREM-1 and MIF had limited value as single markers, whereas PCT and CRP exhibited acceptable diagnostic characteristics.

**Trial registration:**

NCT 00389337

## Introduction

Bacterial infections and sepsis are major causes of morbidity and mortality in medical departments and intensive care units (ICUs) [1-3]. Accurate and timely diagnosis of infection remains challenging to both clinician and laboratory. Clinical and laboratory signs of systemic inflammation, including changes in body temperature, tachycardia, respiratory rate and leucocytosis, are sensitive. However, their use is limited by poor specificity for the diagnosis of sepsis, because critically ill patients often present with the systemic inflammatory response syndrome (SIRS) but no infection [1,4-6]. These issues have fuelled the search for a reliable marker. Many potential biomarkers have been investigated, but only C-reactive protein (CRP) and procalcitonin (PCT) are currently used on a routine basis [7-10]. The search for a single magic bullet marker might ultimately be fruitless, but a combination of markers could improve diagnosis, prognosis and treatment efficacy, and thereby survival [7].

A recently discovered biomarker, soluble triggering receptor expressed on myeloid cells (sTREM)-1, is known to be upregulated on phagocytic cells in the presence of bacteria or fungi [11]. sTREM-1 has been found to be more sensitive and specific than both CRP and PCT in diagnosing sepsis in ICU patients with SIRS [12,13]. The value of sTREM-1 in diagnosing sepsis in settings other than the ICU remains to be determined. Another novel infectious disease biomarker is soluble urokinase-type plasminogen activator receptor (suPAR). Concentrations of suPAR are increased in conditions that involve immune activation, and studies have shown that high concentrations of suPAR portend a poor clinical outcome in diverse infections such as tuberculosis, malaria and pneumococcal bacteraemia [14,15]. Finally, the cytokine macrophage migration inhibitory factor (MIF) has been found to be a valuable marker of microbiologically documented infection in patients who have undergone cardiac surgery [16], and elevated MIF concentrations may be an early indicator of poor outcome in patients with sepsis [17]. The use of sTREM-1, suPAR and MIF to diagnose community-acquired bacterial infections in medical patients has not yet been studied.

We undertook the present study to determine the discriminative power of combining multiple markers to diagnose bacterial infections in adult medical patients admitted to a hospital who are suspected of having community-acquired infections.

## Materials and methods

### Participants

This prospective observational study was conducted from February 2005 to February 2006 at an 800-bed university hospital. All consecutive newly admitted (< 24 hours) adult patients (age ≥ 18 years), who fulfilled at least two criteria for SIRS [6] and who were admitted to the Department of Infectious Diseases or the infectious disease unit in Medical Emergency Department, were asked to participate.

The principal investigator and study nurses recruited patients and collected data on two daily rounds on each week day. Based on data obtained during week days, it was estimated that during the entire study period about 1,800 patients were admitted to the Department of Infectious Diseases from home and that 33% of admitted patients fulfilled at least two SIRS criteria. Of these, 59% were ineligible to participate for the following reasons: admission > 24 hours before evaluation or referral from other departments/hospitals (24%), failure to provide informed written consent (22%), age under 18 years (5.2%), refusal to participate (2.6%), and other reasons (for instance, communication problems; 3.7%). All evaluable patients were included in the main analysis.

The only protocol-driven procedures were blood sampling, collection of data for later calculation of admission Simplified Acute Physiology Scale II and Sequential Organ Failure Assessment scores [18,19], and daily recording of temperature, pulse rate, blood pressure and respiratory rate over one week. Mortality rates at 30 days and 6 months after inclusion were recorded by accessing the Danish Civil Registration System and patient charts. Blood was drawn from a cubital vein into Vacutainer tubes (Becton Dickinson, Plymouth, UK) directly after patient inclusion. The sampling followed routine hospital procedures and was performed by biotechnicians. Plasma from one 6 ml K2-EDTA coated tube was separated by centrifugation and stored at -20°C for up to one week and then transferred to -80°C for later analysis of PCT, suPAR, sTREM-1 and MIF.

The Scientific Ethical Committee of Copenhagen and Frederiksberg Communes approved sample collection on the basis of informed written consent (KF01-108/04). The study protocol is registered on the internet (NCT00389337) [20].

### Reference standard

All patients were grouped into one of the following four groups: no infection present, bacterial infection, viral infection, or parasitic infection. Classification was based on clinical findings, on laboratory findings, response to treatments, radiographic and other imaging procedures, and both positive and negative bacteriological, viral and parasitic findings (including culture, polymerase chain reaction, serological and antigen tests performed) during the first seven days of admission. An expert panel consisting of two infectious disease specialists (OA and GK) retrospectively reviewed all medical records pertaining to each patient and independently decided on the diagnosis at the time of admission. The precise weighting of each finding was greatly dependent on the disease diagnosed (for instance, chest radiography in the diagnosis of respiratory tract infections and cerebrospinal fluid cell counts in the case of viral meningitis). Disagreement among reviewers was discussed, and agreement was reached by consensus. The panel was blinded to PCT, suPAR, sTREM-1 and MIF values, and was instructed to disregard CRP levels and neutrophil counts.

### Test methods

Duplicate measurements of plasma suPAR, sTREM-1 and MIF were performed using a Luminex (Luminex corp. Austin, TX, USA) multiplex assay, as described in detail previously [21]. Margins of error for suPAR, sTREM-1 and MIF measurements are 10%, 12% and 13%, respectively. PCT plasma concentrations were measured using an automated sandwich immunoassay based on the TRACE (time-resolved amplified cryptate emission) technique, in accordance with the manufacturer's protocol (Kryptor; Brahms Diagnostica, Berlin-Henningsdorf, Germany). CRP was measured in plasma by standard densiometry (Vitros 950 IRC; Johnson & Johnson, Clinical Diagnostics Inc., Rochester, NY, USA). Margins of error for both the PCT and CRP assays are 10%. Blood leucocyte and neutrophil counts were measured using the Avida 120 device (Bayer Diagnostics, Tarrytown, NY, USA). Margins of error for these measures were 3.3% and 4.8%, respectively. The principal investigator conducted the Luminex multiplex assay; the Kryptor assay was conducted by one laboratory technician; and the CRP and leucocyte assays were conducted by the hospital laboratory technicians who were on duty when patients were enrolled in the study.

Before the study we chose to use cutoff values of 60 mg/l, 0.25 μg/l and 7.5 × 10^9 ^cells/l for CRP, PCT and neutrophils, respectively. The cutoffs were based on previously reported findings from cohorts similar to the present one [22-25]. Optimal sTREM-1, suPAR, MIF, and three-marker and six-marker cutoff values were determined using Youdens Index [26], because of a lack of reference literature. Laboratory parameters included in the Simplified Acute Physiology Scale II and Sequential Organ Failure Assessment scores were analyzed at the Department of Clinical Biochemistry, Copenhagen University hospital, Hvidovre, Denmark and followed routine procedures.

### Statistics

Measurements of suPAR, sTREM-1, MIF, CRP and PCT were transformed using the logarithmic function in order to obtain normality of distribution within disease groups. Neutrophil count was not transformed. The Mann-Whitney *U*-test was used to compare concentrations of all single markers in patients with documented bacterial infections with those in patients who had undocumented bacterial infections. Sensitivities and specificities with precise 95% confidence intervals (CIs) were calculated for all single and composite markers [27]. Information from the three single best performing markers and all six markers were combined using the method reported by by Xiong and coworkers [27], that is, by identifying the linear combination of markers that yielded the greatest area under the receiver operating characteristic (ROC) curve (AUC). This led to the construction of a composite three-marker test and a composite six-marker test optimized to differentiate between bacterial and nonbacterial causes of inflammation. Standard errors of the AUCs were obtained using the method reported by Xiong and coworkers [27], based on Fisher's Z transformation. The diagnostic performances of the composite markers were compared with the performances of all singles marker using the AUC, in accordance with by the method suggested by Hanley and McNeil [28]. All tests were two sided, and *P *< 0.05 was considered statistically significant. Data were analyzed using the statistical package R version 2.3.1 (R Development Core Team, Vienna, Austria). Figures were drawn using GraphPad Prism version 4.01 (GraphPad Software, San Diego, CA, USA).

## Results

A total of 161 patients fulfilling at least two SIRS criteria were included in the study. Because of exceeded time limits between admission and the index test, non-evaluable samples, missing data and withdrawal of consent, 10 patients were subsequently excluded. For the remaining 151 patients, clinical and demographic characteristics, comorbidity and antibiotic treatment before admission are summarized in Table 1.

**Table 1:** Baseline characteristics

Characteristic	Patients (%; *n *= 151)
Age (years; median [range])	56 (20–94)
Sex	
Male	73 (48.3)
Female	78 (51.7)
Comorbidity^a^	67 (44.7)
Solid tumours and haematological malignancies	14 (9.3)
HIV infection	17 (11.3)
Diabetes	13 (8.6)
COPD and asthma	15 (9.9)
Drug or alcohol abuse	13 (8.6)
Other diseases^b^	17 (11.3)
Medication before admission	
Bacterial antibiotics	39 (25.8)
Immunosuppressives^c^	9 (6.0)
Disease severity	
SAPS II (median [5th to 95th percentile])	18 (6–36)
SOFA score	
0–1	86 (57.0)
2–3	48 (31.8)
4–5	12 (7.9)
>5	5 (3.3)

Data are expressed as *n *(%), unless otherwise indicated. ^a^Several patients had more than one comorbidity (for eample, three had both HIV infection and viral hepatitis). ^b^Inflammatory bowl disease, rheumatoid arthritis, disseminated sclerosis, chronic adrenal insufficiency, viral hepatitis, cardio vascular diseases, and diseases of the thyroid gland. ^c^Steroids, methotrexate, azathioprine, and monoclonal tumour necrosis factaor-α antibodies. COPD, chronic obstructive pulmonary disease; SAPS, Simplified Acute Physiology Score; SOFA, Sepsis-related Organ Failure Assessment.

The expert panel classified 117 patients as infected: 96 with a bacterium, 16 with a virus and five with a parasite. From all but three patients, blood cultures were obtained at admission. A pathogenic bacterium was isolated from blood in 22 patients (15%). At admission and during the first seven days in the hospital, additional cultures were conducted in urine from 96 (64%), sputum from 57 (38%), swabs (skin, wound, or mucosal) from 22 (15%), stools from 19 (13%), and cerebrospinal fluid from 13 (8.6%) patients. A clinically relevant pathogen was isolated from 74 (49%) of the patients. Primary sites of infection and pathogens isolated are summarized in Table 2. All 19 patients classified as having a bacterial infection in the respiratory system in the absence of microbial documentation had chest radiograph findings suggestive of bacterial infection. In the 34 patients classified as non-infected, the causes of SIRS were respiratory distress (lung oedema, chronic obstructive pulmonary disease (COPD) exacerbation with no signs of infection, and embolus of the lung; (*n *= 8), malignant disease (*n *= 8), intracranial haemorrhage (*n *= 2), allergic reaction (*n *= 2), metabolic acidosis (*n *= 2), noninfectious pancreatitis (*n *= 1), gout (*n *= 1), use of impure intravenous drugs (*n *= 1), ruptured mitral valve chordae (*n *= 1), ruptured thoracic aneurism (*n *= 1), Castleman's disease (*n *= 1), Addison's disease (*n *= 1), subileus (*n *= 1) and polymyositis (*n *= 1). Finally, in three patients no explanation for SIRS was found. There was disagreement among reviewers in 11 cases; by consensus, seven of these were classified as non-infected, two as bacterial infection and two as viral infection.

**Table 2:** Site of infection and pathogens isolated

Site of infection (*n*)^a^	Pathogens isolated (*n*)^a^
Respiratory system (58)	*Streptococcus pneumonia *(14), *Legionella pneumonia *(4), *Mycobacterium tuberculosis *(3), *Haemophilus influenza *(3), *Moraxella catarrhalis *(2), *Mycoplasma pneumonia *(2), *Pseudomonas aeruginosa *(1), *Chlamydia psittaci *(1), *Escherichia coli *(1), *Streptococcus haemolytica *group A (1), varicella zoster virus (1), coronavirus (1), unknown bacterial^b ^(19), unknown viral^b ^(5)

Urinary tract (25)	*Escherichia coli *(19), *Streptococcus haemolytica *group G (1), unknown bacterial^b ^(5)

Gastrointestinal tract (16)	*Campylobacter jejuni *(3), *Salmonella enteritidis *(2), *Bacteroides fragilis *(1), *Salmonella dublin *(1), *Salmonella typhi *(1), *Streptococcus haemolytica *group C (1), rotavirus (1), unknown bacterial^b ^(4), unknown viral^b ^(2)

Skin/soft tissue and bone/joint infection (8)	*Streptococcus haemolytica *groups B and G (2), *Staphylococcus aureus *(1), unknown bacterial^b ^(4), unknown viral^b ^(1)

Cenral nervous system (5)	*Neisseria meningitidis *(1), *Streptococcus pneumoniae *(1), unknown viral^b ^(3)

Miscellaneous (9)	*Trepomena palidum *(1), *Enterococcus gallinarum *(1), *Plasmodium falciparum *(5), Epstein-Barr virus (2)

Data are expressed as number of patients (in parenttheses). ^a ^Four patients had two sites of infection; two had pneumonia and urinary tract infection, one had meningitis and pneumonia, and one had staphylococcal skin infection and malaria. ^b^Classified by two specialists in infectious diseases based on typical clinical presentation, anamnesis, chest radiography and other imaging, and cell counts from culture-negative pleura fluid, urine, and cererospinal fluid. Consensus was achieved in all cases.

**Figure 1:**
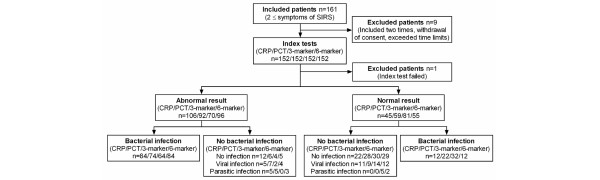
Flowchart of the patients included in the study. Flowchart describing the number of patients included in the study, the reasons for subsequent exclusions, the final diagnoses of the patients, and the ability C-reactive protein (CRP), procalcitonin (PCT), and the three-marker and six-marker combined tests to correctly diagnose patients as having bacterial infection. Optimal cutoffs for bacterial infection (determined by Youdens Index) were used for all four markers. SIRS, systemic inflammatory response syndrome.

We compared concentrations of the various markers between the 64 patients with documented bacterial infection and the 32 patients classified as having bacterial infection of unknown origin. The respective median concentrations were as follows: 175 and 157.5 mg/l (*P *= 0.70) for CRP, 0.96 and 0.87 μg/l (*P *= 0.26) for PCT, 11.0 and 10.6 × 10^9 ^cells/l (*P *= 0.81) for neutrophils, 2.4 and 2.3 μg/l (*P *= 0.77) for suPAR, 7.9 and 8.5 μg/l (*P *= 0.36) for sTREM-1, and 1.4 and 1.3 μg/L (*P *= 0.86) for MIF. Recruitment, exclusion and subsequent grouping of all patients included in the study are shown in Figure 1.

A total of 120 patients (79%) were given antibiotics during the first 24 hours of hospitalization: 64% of the patients with inflammation of nonbacterial origin and 90% of the patients with a bacterial infection. Six patients without a bacterial infection (11%) and three (3.1%) with a bacterial infection died before day 30 after admission. After six months, 11 (20%) patients who did not have a bacterial infection and eight (8.3%) patients who did have a bacterial infection had died.

**Figure 2:**
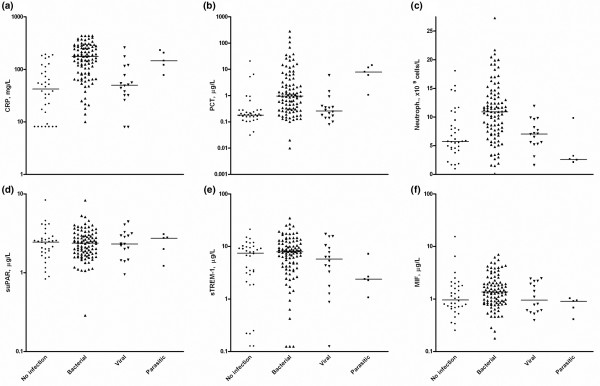
Plasma concentrations of the markers. Shown are individual admission plasma concentrations of **(a) **C-reactive protein (CRP), **(b) **procalcitonin (PCT), **(c) **neutrophil count, **(d) **soluble urokinase-type plasminogen activator receptor (suPAR), **(e) **soluble triggering receptor expressed on myeloid cells (sTREM)-1 and **(f) **macrophage migration inhibitory factor (MIF) in patients with no infection (circle), bacterial (triangle, apex up), viral (triangle, apex down), or parasitic infection (square). Bars represent the medians of the concentrations.

**Figure 3:**
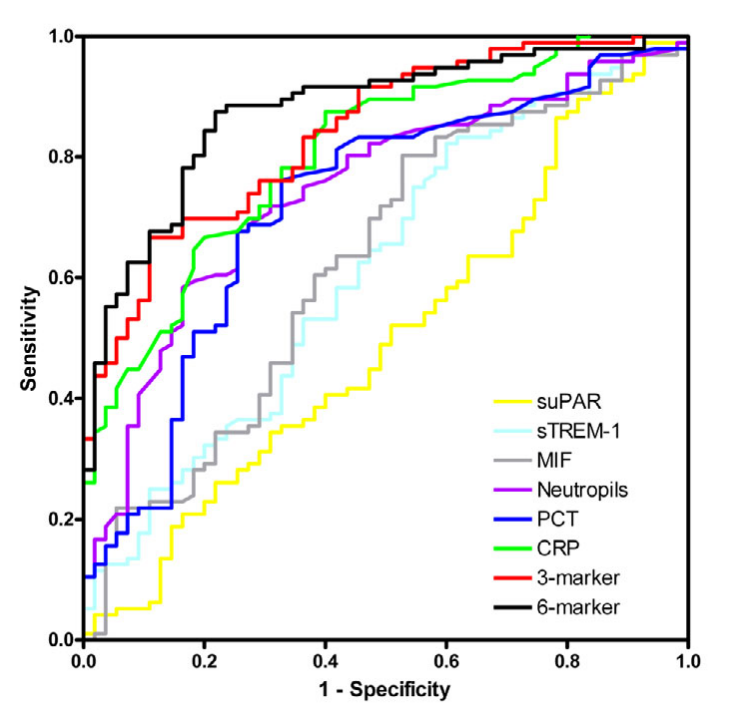
ROC curves comparing markers' ability to detect bacterial infections in patients with systemic inflammation. Receiver operating characteristic (ROC) curves comparing soluble urokinase-type plasminogen activator receptor (suPAR), soluble triggering receptor expressed on myeloid cells (sTREM)-1, macrophage migration inhibitory factor (MIF), neutrophil count, procalcitonin (PCT), C-reactive protein (CRP), and the combined three-marker and six-marker tests for detection of bacterial versus nonbacterial causes of systemic inflammation.

Individual baseline values and median levels of the six biomarkers are shown in Figure 2. The computed specificities, sensitivities, positive and negative predictive values, and AUCs of the single markers and the composite markers with regard to diagnosis of bacterial infection are shown in Table 3. The corresponding ROC curves are shown in Figure 3. The six-marker test performed significantly better than all of the single markers (*P *= 0.010 for CRP and *P *< 0.001 for the five remaining markers). Additional analysis of the ability of single markers to discriminate between infection of any kind and no infection identified AUCs of 0.80 (95% CI 0.71–0.86) for CRP, 0.77 (95% CI 0.67–0.84) for PCT, 0.68 (95% CI 0.57–0.76) for neutrophils, 0.59 (95% CI 0.48–0.70) for MIF, 0.56 (95% CI 0.45–0.67) for sTREM-1 and 0.51 (95% CI 0.40–0.63) for suPAR.

**Table 3:** Accuracy of the six inflammatory markers and the combined three-marker and three-marker tests in diagnosing bacterial infection in SIRS patients

Biomarker	Sensitivity (95% CI)^a^	Specificity (95% CI)^a^	AUC (95% CI)	Specificity = 0.7	Specificity = 0.8	Positive predictive value^b^	Negative predictive value^b^
						
				Sensitivity (95% CI)	Sensitivity (95% CI)		
CRP	0.86 (0.78–0.93)	0.60 (0.46–0.73)	0.81 (0.73–0.86)	0.72 (0.62–0.81)	0.67 (0.56–0.76)	0.79	0.73
PCT	0.80 (0.71–0.88)	0.58 (0.44–0.71)	0.72 (0.63–0.79)	0.69 (0.58–0.78)	0.51 (0.41–0.61)	0.80	0.63
Neutrophil count	0.74 (0.64–0.82)	0.64 (0.50–0.76)	0.74 (0.66–0.81)	0.70 (0.60–0.79)	0.59 (0.49–0.69)	0.82	0.57
MIF	0.80 (0.71–0.88)	0.47 (0.34–0.61)	0.63 (0.53–0.72)	0.41 (0.31–0.51)	0.29 (0.20–0.39)	0.73	0.58
sTREM-1	0.82 (0.73–0.89)	0.40 (0.27–0.54)	0.61 (0.52–0.71)	0.36 (0.27–0.47)	0.32 (0.23–0.43)	0.71	0.56
suPAR	0.35 (0.26–0.46)	0.67 (0.53–0.79)	0.50 (0.40–0.60)	0.31 (0.22–0.42)	0.23 (0.15–0.33)	0.65	0.37
3-marker^c^	0.67 (0.56–0.76)	0.89 (0.78–0.96)	0.84 (0.71–0.91)	0.76 (0.66–0.84)	0.70 (0.60–0.79)	0.91	0.60
6-marker^d^	0.88 (0.79–0.93)	0.78 (0.65–0.88)	0.88 (0.81–0.92)	0.89 (0.80–0.94)	0.84 (0.76–0.91)	0.88	0.78

^a^Sensitivity and specificity of C-reactive protein (CRP), procalcitonin (PCT) and neutrophil count were computed using the predefined cutoff values of 60 mg/l, 0.25 μg/l and 7.5 × 10^9 ^cells/l, respectively. Sensitivity and specificity of macrophage migration inhibitory factor (MIF), soluble triggering receptor expressed on myeloid cells (sTREM)-1, soluble urokinase-type plasminogen activator receptor (suPAR), and the three-marker and six-marker tests were computed using optimal cutoff values determined using Youdens Index. ^b^Positive and negative predictive values were calculated using Youdens Index-determined optimal cutoffs for all markers. The optimal cutoffs were 59 mg/l for CRP, 0.28 μg/l for PCT, 8.5 × 10^9 ^cells/l for neutrophil count, 0.81 μg/l for MIF, 3.5 μg/l for sTREM-1, 2.7 μg/l for suPAR, 6.1 for the three-marker test and 4.1 for the six-marker test. ^c^Three-marker test = 0.160 × neutrophil count + 0.981 × log(CRP) + 0.107 × log(PCT). ^d^Six-marker test = -0.551 × log(suPAR) + 0.254 × log(sTREM-1) + 0.416 × log(MIF) + 0.098 × neutrophils + 0.639 × log(CRP) + 0.201 × log(PCT). AUC, area under the receiver operating characteristic curve; CI confidence interval.

It was apparent from Figure 2 that patients with a parasitic (*Plasmodium falciparum*) infection had high concentrations of CRP and PCT in particular, and so the effect of omitting these patients on the AUCs for these two markers was determined. This analysis identified AUCs of 0.83 (95% CI 0.76–0.90) and 0.77 (95% CI 0.69–0.85) for CRP and PCT, respectively, with regard to discrimination between bacterial and nonbacterial causes of inflammation. Several of the markers may be affected by immune-deficient conditions, and therefore an ancillary analysis was conducted in which 38 patients with solid tumours, haematological malignancies, HIV infection, leucocyte counts below 1 × 10^9 ^cells/l, or treated with an immunosuppressant were excluded. In this analysis the ability of the markers to diagnose bacterial infections remained virtually unchanged. None of the single marker AUCs changed by more than 0.04 (data not shown).

To investigate the diagnostic accuracy of the six single markers and the two composite markers in a relevant subgroup, an analysis of the 57 patients diagnosed as having COPD or asthma with acute exacerbation or pneumonia (excluding *Mycobacterium tuberculosis *infection) was performed. With respect to the diagnosis of bacterial infection we obtained AUCs of 0.94 (95% CI 0.87–1.00) for the six-marker test, 0.88 (95% CI 0.78–0.97) for the three-marker test, 0.88 (95% CI 0.79–0.97) for CRP, 0.79 (95% CI 0.67–0.91) for PCT, 0.76 (95% CI 0.62–0.91) for sTREM-1, 0.72 (95% CI 0.56–0.89) for neutrophils, 0.66 (95% CI 0.47–0.85) for MIF and 0.54 (95% CI 0.34–0.74) for suPAR.

In addition, the ability of single markers to predict culture-proven bacteraemia was tested. The three markers with the greatest AUCs were PCT, CRP and MIF, with AUCs of 0.84 (95% CI 0.70–0.92), 0.69 (95% CI 0.54–0.80) and 0.61 (95% CI 0.46–0.72), respectively.

## Discussion

In the present study, we demonstrate that there is a significant gain in discriminative power of diagnostic sepsis markers when the linear combination that yields the highest AUC is employed. In addition, in patients admitted to a medical emergency department or a department of infectious diseases, we found that sTREM-1, MIF and suPAR as single markers have limited diagnostic power to discriminate between bacterial and nonbacterial causes of inflammation. However, if they are combined with CRP, PCT and neutrophil count a high AUC of 0.88 is achieved.

The majority of studies of new sepsis biomarkers examine these biomarkers one at a time. Measurements of plasma concentrations of each putative marker with individual assays carry considerable burdens in terms of time, cost and sample volume, thus limiting ability to examine systematically the potential of multiple markers in combination. However, xMAP technology provides the possibility to quantify multiple proteins simultaneously in a solution phase using flow cytometry [21]. This allows the researcher to profile multiple markers for diagnostic and prognostic purposes simultaneously, and to monitor changes over time in the markers to evaluate the efficacy of treatment.

Having techniques to measure multiple markers simultaneously and being presented with a complex diagnostic challenge such as sepsis raises another question; how does one optimally combine information from multiple markers? The power of combining multiple sepsis markers is recognized, but earlier studies used informal and suboptimal quantitative approaches to identify the optimal combination. Several statistical studies have addressed the problem of combining correlated diagnostic tests to maximize discriminatory power. These include logistic regression and linear and nonlinear discriminate analyses to identify the linear combination of markers that yield the greatest AUC [29,30]. These models derive a score but not a specific decision rule, as decision trees, Bayesian decision making and neural networks do [4,27,29,31-35].

The combination of diagnostic markers appears a useful approach to improving accuracy in diagnosing sepsis in patients with SIRS and may be applicable to other complex diseases as well. Use of ROC curves and comparison of AUCs for single markers has become widespread; however, although the statistical techniques needed to identify the combination of ROC curves from multiple markers that yield the greatest AUC have been available for some years, there use has been limited. Only few studies have applied the statistical techniques developed by Su and Liu [27,34]. These found increased accuracy when diagnostic test were combined to diagnose Alzheimer's disease and prostate cancer, respectively.

However, it is important to remember that the hunt for a larger AUC might not always be clinically relevant. This is the case if the gain is associated with very low sensitivity or specificity, as was observed in our study, in which the sensitivity of PCT at the predefined clinically relevant specificities was second highest; only the six-marker test had higher sensitivity. In comparison the AUC of PCT was lower than both the AUCs of the six-marker test, the three-marker test and CRP.

Promising results with sTREM-1 as a diagnostic sepsis marker were reported over recent years [12,13,36]. Gibot and coworkers [13] measured sTREM-1 in plasma samples from ICU patients with SIRS suspected of having an infection; they found that sTREM-1 was able to diagnose infection with a sensitivity of 96% (95% CI 92–100%) and a specificity of 89% (95% CI 82–95%). There were large difference between the two patient cohorts, both in terms of spectrum and severity of disease. It is known from previous studies that the diagnostic accuracies of several sepsis markers are highly dependent on the setting in which they are tested. Based on data from these studies, it seems that PCT, in particular, exhibits superior performance to that of CRP when it is used in an ICU; this might as well be the case for sTREM-1 [3,9,13,22,25,37-43]. In addition, different analytical methods, plasma anticoagulants, and plasma sampling and processing procedures were used [12,21]. In this regard we have shown that the half-life of sTREM-1 in plasma is short (1.5 hours), and so our handling procedures in the present study might have been too slow [21]. Recently published findings on plasma sTREM-1 in patients with pneumonia, COPD and asthma in a setting similar to ours indicate no difference in admission levels of sTREM-1 between COPD and pneumonia patients, although the AUC for guidance of antibiotic therapy was found to be 0.77 (95% CI 0.70–0.84) [44], which is almost identical to the AUC of 0.76 (95% CI 0.62–0.91) achieved in our subgroup analysis. Other interesting findings are that in patients with inflammatory bowel disease a 400-fold increase in sTREM-1 concentration was observed in those with severe disease as compared with patients with only mild symptoms [45]. Also, in a murine air-pouch model of crystal-induced acute inflammation, monosodium urate monohydrate crystals induced high concentrations of sTREM-1 [46]. Based on the present data on sTREM-1 as a marker of infection, it seems reasonable to conclude that more studies, using the same meticulously validated assay and in more clinically relevant patient groups, are needed.

Studies investigating the use of PCT and CRP in medical and emergency departments have found the diagnostic performance of CRP and PCT to be similar to those observed in our study [22,25,37]. With regard to diagnosing bacteraemia in particular, PCT exhibited excellent diagnostic ability; this is in accordance with the suggested notion that PCT is superior to CRP in diagnosing systemic infection [22,37,47,48]. The low diagnostic accuracy of PCT in diagnosing bacterial infection observed in our study was partly due to the five patients infected with *P. falciparum*, as was shown in the analysis in which this group was omitted.

Despite our study's strengths, however, several limitations deserve consideration. It is probably an oversimplification to use a linear model to combine markers. Quadratic or cubic transformations of the biomarkers might improve diagnostic accuracy. Because we used clinical criteria and microbiological evidence, it might have been difficult to ascertain the precise cause of SIRS in all patients, and this might have introduced some misclassification bias. The expert panel disregarded measurements of leucocytes and CRP, but – as in most studies on diagnostic sepsis markers – total blinding was not achievable, because these measurements are an integrated part the routine monitoring of infectious disease patients and the values are reflected in the way in which the patient is treated. This might have lead to incorporation bias and thus an overestimation of the diagnostic power of these two markers as compared with the other markers tested, although this was not reflected in any statistically significant differences in the concentrations of any of the markers in the patients with 'known' versus 'unknown' bacterial infection. Thus, it seems that no marker was afforded preferential conditions by the classification. The fact that not all samples were collected before antibiotic therapy was initiated might weaken the results, because markers with short half-life would be more affected than markers with long half-life. Patients with dementia or other mental diseases could not participate in this study (because of the need for informed written consent), and so it is not know whether the results are valid for this important group of patients. Finally, our results may apply only to patients with community-acquired infections, which do not require hospitalization in an ICU directly at admission, and so they may not be valid in ICU patients.

## Conclusion

Our results demonstrate that combining information from several sepsis markers is simple and may significantly improve clinicians' ability to differentiate patients with bacterial infections from those with systemic inflammation of nonbacterial origin when they are admitted. This would be of great importance in patients in whom diagnosis is not clinically clear cut, as is often the case in a specialized department of infectious diseases, bearing in mind that rapid and adequate treatment of patients suspected of having bacterial sepsis requires accurate diagnosis.

## Key messages

• Combining information from several markers appears to improve diagnostic accuracy for detection of bacterial versus nonbacterial causes of systemic inflammation.

• In a cohort of patients with SIRS, admitted to a medical emergency department or a department of infectious diseases and suspected of having community-acquired infections, single measurements suPAR, sTREM-1 and MIF appear to have limited power as diagnostic markers for bacterial infection.

• CRP, PCT and neutrophil count have acceptable diagnostic power for the diagnosis of community-acquired bacterial infection in patients with SIRS admitted to a department of infectious diseases.

• The diagnostic accuracy of CRP, PCT, sTREM-1, and the six-marker test was higher in the subgroup of patients suspected of having pneumonia than in the group as a whole.

## Abbreviations

AUC = area under the receiver operating characteristic curve; CI = confidence interval; CRP = C-reactive protein; ICU = intensive care unit; MIF = macrophage migration inhibitory factor; PCT = procalcitonin; ROC = receiver operating characteristic; SIRS = systemic inflammatory response syndrome; SOFA = Sequential Organ Failure Assessment; suPAR = soluble receptors urokinase-type plasminogen activator; sTREM = soluble triggering receptor expressed on myeloid cells.

## Competing interests

suPAR antibodies were a gift from ViroGates (Cape Town, South Africa). JE is a shareholder in ViroGates and holds patents on using suPAR for diagnostic and prognostic purposes.

## Authors' contributions

KK planned the study, wrote the protocol, collected data, carried out the analyses of suPAR, sTREM-1 and MIF, and wrote the manuscript. OA contributed to the concept of the study, the writing of the protocol and the grouping of patients, and helped to draft the manuscript. GK participated in planning of the study and grouping of patients, and helped to draft the manuscript. JE contributed to the planning of the study and the analysis of suPAR, sTREM-1 and MIF. MT was responsible for the analyses of PCT and helped to draft the manuscript. JP was involved in the analyses of data, the construction of the combined markers and drafting of the manuscript. KL participated in design and concept of the study, was responsible for statistical analyses of data, and participated in drafting the manuscript. All authors read and approved the final manuscript.

## References

[B1] Alberti C, Brun-Buisson C, Goodman SV, Guidici D, Granton J, Moreno R, Smithies M, Thomas O, Artigas A, Le Gall JR (2003). Influence of systemic inflammatory response syndrome and sepsis on outcome of critically ill infected patients. Am J Respir Crit Care Med.

[B2] Sands KE, Bates DW, Lanken PN, Graman PS, Hibberd PL, Kahn KL, Parsonnet J, Panzer R, Orav EJ, Snydman DR (1997). Epidemiology of sepsis syndrome in 8 academic medical centers. JAMA.

[B3] Flaatten H (2004). Epidemiology of sepsis in Norway in 1999. Crit Care.

[B4] Jaimes F, Arango C, Ruiz G, Cuervo J, Botero J, Velez G, Upegui N, Machado F (2004). Predicting bacteremia at the bedside. Clin Infect Dis.

[B5] Levy MM, Fink MP, Marshall JC, Abraham E, Angus D, Cook D, Cohen J, Opal SM, Vincent JL, Ramsay G (2003). 2001 SCCM/ESICM/ACCP/ATS/SIS International Sepsis Definitions Conference. Crit Care Med.

[B6] Bone RC, Balk RA, Cerra FB, Dellinger RP, Fein AM, Knaus WA, Schein RM, Sibbald WJ (1992). Definitions for sepsis and organ failure and guidelines for the use of innovative therapies in sepsis. The ACCP/SCCM Consensus Conference Committee. American College of Chest Physicians/Society of Critical Care Medicine. Chest.

[B7] Carrigan SD, Scott G, Tabrizian M (2004). Toward resolving the challenges of sepsis diagnosis. Clin Chem.

[B8] Meisner M (2005). Biomarkers of sepsis: clinically useful?. Curr Opin Crit Care.

[B9] Mitaka C (2005). Clinical laboratory differentiation of infectious versus non-infectious systemic inflammatory response syndrome. Clin Chim Acta.

[B10] Marshall JC, Vincent JL, Fink MP, Cook DJ, Rubenfeld G, Foster D, Fisher CJ, Faist E, Reinhart K (2003). Measures, markers, and mediators: toward a staging system for clinical sepsis. A report of the Fifth Toronto Sepsis Roundtable, Toronto, Ontario, Canada, October 25–26, 2000. Crit Care Med.

[B11] Colonna M, Facchetti F (2003). TREM-1 (triggering receptor expressed on myeloid cells): a new player in acute inflammatory responses. J Infect Dis.

[B12] Gibot S, Cravoisy A, Levy B, Bene MC, Faure G, Bollaert PE (2004). Soluble triggering receptor expressed on myeloid cells and the diagnosis of pneumonia. N Engl J Med.

[B13] Gibot S, Kolopp-Sarda MN, Bene MC, Cravoisy A, Levy B, Faure GC, Bollaert PE (2004). Plasma level of a triggering receptor expressed on myeloid cells-1: its diagnostic accuracy in patients with suspected sepsis. Ann Intern Med.

[B14] Eugen-Olsen J, Gustafson P, Sidenius N, Fischer TK, Parner J, Aaby P, Gomes VF, Lisse I (2002). The serum level of soluble urokinase receptor is elevated in tuberculosis patients and predicts mortality during treatment: a community study from Guinea-Bissau. Int J Tuberc Lung Dis.

[B15] Wittenhagen P, Kronborg G, Weis N, Nielsen H, Obel N, Pedersen SS, Eugen-Olsen J (2004). The plasma level of soluble urokinase receptor is elevated in patients with *Streptococcus pneumoniae *bacteraemia and predicts mortality. Clin Microbiol Infect.

[B16] Mendonca-Filho HT, Gomes GS, Nogueira PM, Fernandes MA, Tura BR, Santos M, Castro-Faria-Neto HC (2005). Macrophage migration inhibitory factor is associated with positive cultures in patients with sepsis after cardiac surgery. Shock.

[B17] Bozza FA, Gomes RN, Japiassu AM, Soares M, Castro-Faria-Neto HC, Bozza PT, Bozza MT (2004). Macrophage migration inhibitory factor levels correlate with fatal outcome in sepsis. Shock.

[B18] Le Gall JR, Lemeshow S, Saulnier F (1993). A new Simplified Acute Physiology Score (SAPS II) based on a European/North American multicenter study. JAMA.

[B19] Vincent JL, Moreno R, Takala J, Willatts S, De Mendonca A, Bruining H, Reinhart CK, Suter PM, Thijs LG (1996). The SOFA (Sepsis-related Organ Failure Assessment) score to describe organ dysfunction/failure. On behalf of the Working Group on Sepsis-Related Problems of the European Society of Intensive Care Medicine. Intensive Care Med.

[B20] Sepsis: Prognosis and Evaluation of Early Diagnosis and Intervention (SPEEDI Study). http://www.clinicaltrials.gov/ct/show/NCT00389337?order=1.

[B21] Kofoed K, Schneider UV, Scheel T, Andersen O, Eugen-Olsen J (2006). Development and validation of a multiplex add-on assay for sepsis biomarkers using xMAP technology. Clin Chem.

[B22] Chan YL, Tseng CP, Tsay PK, Chang SS, Chiu TF, Chen JC (2004). Procalcitonin as a marker of bacterial infection in the emergency department: an observational study. Crit Care.

[B23] Christ-Crain M, Jaccard-Stolz D, Bingisser R, Gencay MM, Huber PR, Tamm M, Muller B (2004). Effect of procalcitonin-guided treatment on antibiotic use and outcome in lower respiratory tract infections: cluster-randomised, single-blinded intervention trial. Lancet.

[B24] Davis BH, Bigelow NC (2005). Comparison of neutrophil CD64 expression, manual myeloid immaturity counts, and automated hematology analyzer flags as indicators of infection or sepsis. Lab Hematol.

[B25] Gaini S, Koldkjaer OG, Pedersen C, Pedersen SS (2006). Procalcitonin, lipopolysaccharide-binding protein, interleukin-6 and C-reactive protein in community-acquired infections and sepsis: a prospective study. Crit Care.

[B26] Youden WJ (1950). Index for rating diagnostic tests. Cancer.

[B27] Xiong C, McKeel DW, Miller JP, Morris JC (2004). Combining correlated diagnostic tests: application to neuropathologic diagnosis of Alzheimer's disease. Med Decis Making.

[B28] Hanley JA, McNeil BJ (1983). A method of comparing the areas under receiver operating characteristic curves derived from the same cases. Radiology.

[B29] McIntosh MW, Pepe MS (2002). Combining several screening tests: optimality of the risk score. Biometrics.

[B30] Su JQ, Liu JS (1993). Linear combinations of multiple diagnostic markers. J Am Stat Assoc.

[B31] Bates DW, Sands K, Miller E, Lanken PN, Hibberd PL, Graman PS, Schwartz JS, Kahn K, Snydman DR, Parsonnet J (1997). Predicting bacteremia in patients with sepsis syndrome. Academic Medical Center Consortium Sepsis Project Working Group. J Infect Dis.

[B32] Harbarth S, Holeckova K, Froidevaux C, Pittet D, Ricou B, Grau GE, Vadas L, Pugin J (2001). Diagnostic value of procalcitonin, interleukin-6, and interleukin-8 in critically ill patients admitted with suspected sepsis. Am J Respir Crit Care Med.

[B33] Paul M, Andreassen S, Nielsen AD, Tacconelli E, Almanasreh N, Fraser A, Yahav D, Ram R, Leibovici L (2006). Prediction of bacteremia using TREAT, a computerized decision-support system. Clin Infect Dis.

[B34] Pepe MS, Thompson ML (2000). Combining diagnostic test results to increase accuracy. Biostatistics.

[B35] Peres BD, Melot C, Lopes FF, Vincent JL (2003). Infection Probability Score (IPS): A method to help assess the probability of infection in critically ill patients. Crit Care Med.

[B36] Richeldi L, Mariani M, Losi M, Maselli F, Corbetta L, Buonsanti C, Colonna M, Sinigaglia F, Panina-Bordignon P, Fabbri LM (2004). Triggering receptor expressed on myeloid cells: role in the diagnosis of lung infections. Eur Respir J.

[B37] Hausfater P, Garric S, Ayed SB, Rosenheim M, Bernard M, Riou B (2002). Usefulness of procalcitonin as a marker of systemic infection in emergency department patients: a prospective study. Clin Infect Dis.

[B38] Munoz P, Simarro N, Rivera M, Alonso R, Alcala L, Bouza E (2004). Evaluation of procalcitonin as a marker of infection in a nonselected sample of febrile hospitalized patients. Diagn Microbiol Infect Dis.

[B39] Selberg O, Hecker H, Martin M, Klos A, Bautsch W, Kohl J (2000). Discrimination of sepsis and systemic inflammatory response syndrome by determination of circulating plasma concentrations of procalcitonin, protein complement 3a, and interleukin-6. Crit Care Med.

[B40] Simon L, Gauvin F, Amre DK, Saint-Louis P, Lacroix J (2004). Serum procalcitonin and C-reactive protein levels as markers of bacterial infection: a systematic review and meta-analysis. Clin Infect Dis.

[B41] Uzzan B, Cohen R, Nicolas P, Cucherat M, Perret GY (2006). Procalcitonin as a diagnostic test for sepsis in critically ill adults and after surgery or trauma: a systematic review and meta-analysis. Crit Care Med.

[B42] Muller B, Becker KL, Schachinger H, Rickenbacher PR, Huber PR, Zimmerli W, Ritz R (2000). Calcitonin precursors are reliable markers of sepsis in a medical intensive care unit. Crit Care Med.

[B43] BalcI C, Sungurtekin H, Gurses E, Sungurtekin U, Kaptanoglu B (2003). Usefulness of procalcitonin for diagnosis of sepsis in the intensive care unit. Crit Care.

[B44] Phua J, Koay ES, Zhang DH, Tai LK, Boo XL, Lim KC, Lim TK (2006). Soluble triggering receptor expressed on myeloid cells-1 in acute respiratory infections. Eur Respir J.

[B45] Tzivras M, Koussoulas V, Giamarellos-Bourboulis EJ, Tzivras D, Tsaganos T, Koutoukas P, Giamarellou H, Archimandritis A (2006). Role of soluble triggering receptor expressed on myeloid cells in inflammatory bowel disease. World J Gastroenterol.

[B46] Murakami Y, Akahoshi T, Hayashi I, Endo H, Kawai S, Inoue M, Kondo H, Kitasato H (2006). Induction of triggering receptor expressed on myeloid cells 1 in murine resident peritoneal macrophages by monosodium urate monohydrate crystals. Arthritis Rheum.

[B47] Chirouze C, Schuhmacher H, Rabaud C, Gil H, Khayat N, Estavoyer JM, May T, Hoen B (2002). Low serum procalcitonin level accurately predicts the absence of bacteremia in adult patients with acute fever. Clin Infect Dis.

[B48] Ugarte H, Silva E, Mercan D, De Mendonca A, Vincent JL (1999). Procalcitonin used as a marker of infection in the intensive care unit. Crit Care Med.

